# Autophagy mediates the beneficial effect of hypoxic preconditioning on bone marrow mesenchymal stem cells for the therapy of myocardial infarction

**DOI:** 10.1186/s13287-017-0543-0

**Published:** 2017-04-18

**Authors:** Zheng Zhang, Chao Yang, Mingzhi Shen, Ming Yang, Zhitao Jin, Liping Ding, Wei Jiang, Junke Yang, Haixu Chen, Feng Cao, Taohong Hu

**Affiliations:** 1 Department of Cardiology, The General Hospital of the PLA Rocket Force, Beijing, 100088 China; 2 Department of Blood Transfusion, The General Hospital of the PLA Rocket Force, Beijing, 100088 China; 3 Department of Cardiology, Hainan Branch of PLA General Hospital, Sanya, 572013 China; 4 Department of Cardiology, The General Hospital of Chinese People’s Liberation Army, Beijing, 100853 China; 5 Shanghai Jiao Tong University Affiliated Sixth People’s Hospital, Shanghai, 201306 China; 6 School of Basic Medical Sciences, Taishan Medical University, Taian, Shandong 271000 China; 7 Core Laboratory of Translational Medicine, Institute of Geriatrics, PLA general Hospital, Beijing, 100853 China

**Keywords:** Hypoxic preconditioning, Mesenchymal stem cells, Myocardial infarction, Apoptosis, Autophagy

## Abstract

**Background:**

Stem cell therapy has emerged as a promising therapeutic strategy for myocardial infarction (MI). However, the poor viability of transplanted stem cells hampers their therapeutic efficacy. Hypoxic preconditioning (HPC) can effectively promote the survival of stem cells. The aim of this study was to investigate whether HPC improved the functional survival of bone marrow mesenchymal stem cells (BM-MSCs) and increased their cardiac protective effect.

**Methods:**

BM-MSCs, isolated from Tg*(Fluc-egfp)* mice which constitutively express both firefly luciferase (Fluc) and enhanced green fluorescent protein (eGFP), were preconditioned with HPC (1% O_2_) for 12 h, 24 h, 36 h, and 48 h, respectively, followed by 24 h of hypoxia and serum deprivation (H/SD) injury.

**Results:**

HPC dose-dependently increased the autophagy in BM-MSCs. However, the protective effects of HPC for 24 h are most pronounced. Moreover, hypoxic preconditioned BM-MSCs (^HPC^MSCs) and nonhypoxic preconditioned BM-MSCs (^NPC^MSCs) were transplanted into infarcted hearts. Longitudinal in vivo bioluminescence imaging (BLI) and immunofluorescent staining revealed that HPC enhanced the survival of engrafted BM-MSCs. Furthermore, ^HPC^MSCs significantly reduced fibrosis, decreased apoptotic cardiomyocytes, and preserved heart function. However, the beneficial effect of HPC was abolished by autophagy inhibition with 3-methyladenine (3-MA) and Atg7siRNA.

**Conclusion:**

This study demonstrates that HPC may improve the functional survival and the therapeutic efficiencies of engrafted BM-MSCs, at least in part through autophagy regulation. Hypoxic preconditioning may serve as a promising strategy for optimizing cell-based cardiac regenerative therapy.

**Electronic supplementary material:**

The online version of this article (doi:10.1186/s13287-017-0543-0) contains supplementary material, which is available to authorized users.

## Background

Myocardial infarction (MI) and consequent heart failure remain the leading cause of morbidity and mortality worldwide. The traditional therapies, aimed at preserving the function of the remaining cardiac myocytes, are palliative [1]. Currently, stem cell therapy has emerged as a promising therapeutic strategy to effectively reverse cardiac damage and restore cardiac function in terms of its potential contribution to cardiovascular regeneration [2, 3]. Among these cells, bone marrow mesenchymal stem cells (BM-MSCs), capable of self-renewal and differentiation into various mesenchymal tissues, have been considered as an optimal candidate in regenerative medicine. A large number of studies have demonstrated that BM-MSC transplantation can effectively attenuate myocardial injury and improve cardiac function [4, 5]. In practical applications, however, the therapeutic efficiency of stem cell transplantation is greatly limited by the poor viability and function of donor cells [6]. The harsh host ischemic microenvironment in the ischemic myocardium resulted in high levels of apoptosis, which further impaired the functional survival of transplanted donor stem cells. Moreover, massive cell death not only hampers the efficiency of the therapy, but also introduces an additional burden to the infarcted myocardium [7, 8]. Therefore, optimized strategies that enhance the cell viability and retention of transplanted donor cells are crucial to improving the efficiency of MSC-mediated therapy for MI.

Previous in vitro and in vivo studies have demonstrated that sublethal hypoxic preconditioning (HPC) significantly increases the expressions of pro-survival and pro-angiogenic cellular factors in MSCs [9, 10]. Moreover, HPC increases cell survival, inhibits extensive apoptosis, and thereby improves the adaptability of MSCs to hypoxic injury [11]. Therefore, HPC has been considered as a promising adjuvant strategy in the cell-based treatment for MI. However, the optimal HPC protocol of BM-MSCs for MI therapy remains uncertain. Furthermore, detailed mechanisms underlying the beneficial effects of HPC have not been elucidated.

Autophagy is a highly conserved catabolic process to degrade damaged organelles for recycling of cytoplasmic components by forming autophagosomes, which then fuse with lysosomes to form autolysosomes [12]. The evidence has demonstrated that this can either protect cells or contribute to cell death depending on the different cell types and intensity of stimulus [13]. Autophagy at basal levels is involved in development, differentiation, and maintaining normal function in various organisms. Hence, autophagy has been generally considered as a protective cellular response against various stresses, such as starvation or nutrient depletion. Conversely, previous studies also suggested that extensive and prolonged autophagy may be a promoter of apoptosis, leading to cell death as type II programmed cell death [14]. A significant body of studies has demonstrated that HPC can induce autophagy in MSCs. Moreover, Wang et al. [15] revealed that modest HPC can offer neuroprotection by activating autophagic pathways, indicating a neuroprotective role of autophagy in HPC. Despite expansion of our knowledge, the detailed effect of HPC on autophagy of MSCs has not been fully understood. Accordingly, we hypothesized that the protective effects of HPC on BM-MSCs are mediated by regulating autophagy.

## Methods

### Animals

Adult male C57BL/6 mice were provided by the Animal Center of the Fourth Military Medical University (Xi’an, China). Male L2G85 reporter transgenic mice were purchased from Contag Laboratory (Stanford, CA, USA). L2G85 transgenic mice were created on the FVB background which stably express both firefly luciferase (Fluc) and enhanced green fluorescence protein (eGFP; fLuc-eGFP) in all tissues and organs. Mice were housed in a temperature-controlled animal facility with a 12-h light/dark cycle, with tap water and rodent chow provided ad libitum. All animal studies were performed via a protocol approved by the Animal Care and Use Committee of the Second Artillery General Hospital of PLA (approval ID: 5034) and were in compliance with the Guidelines for the Care and Use of Laboratory Animals, as published by the National Academy Press.

### Isolation, culture, and identification of BM-MSCs from L2G85 transgenic mice

BM-MSCs were isolated and expanded with a modified procedure as described previously [8]. In brief, bone marrow was flushed from the femoral and tibia of adult L2G85 mice with fetal bovine serum (FBS)-free Dulbecco’s modified Eagle’s medium (DMEM). After passing through a 70-μm strainer and centrifugation at 1200 rpm for 5 min at room temperature, the cell pellet was resuspended in DMEM supplemented with 20% FBS and incubated at 37 °C in an atmosphere containing 5% CO_2_. After 24 h, the culture medium was replaced to remove the nonadherent cells, and then was completely replaced every 3 days. Third-passage BM-MSCs with optimal growth at the third generation were used for different treatments to avoid contamination with other cell types.

BM-MSCs were characterized as CD44^+^, CD90^+^, CD34^–^, and CD45^–^ using cytofluorimetric analysis as described previously [8]. Briefly, after being incubated with 1 μL monoclonal PE-conjugated antibodies against specific membrane markers (CD44, CD90, CD34, and CD45; BD, San Jose, CA, USA) for 1 h, BM-MSCs were processed through a FACS Calibur system (BD, San Jose, CA, USA) according to the manufacturer’s protocol. Cells were gated according to their high fluorescence.

Furthermore, the multipotency of BM-MSCs was confirmed by induction of osteogenic and adipogenic differentiation as previously described [8, 16]. Adipogenic medium (alpha-modified Eagles’s medium (αMEM) with 10% fetal calf serum (FCS), 1% antibiotics, 50 μM indomethacin (Sigma), 0.5 mM IBMX (Sigma), and 1 μM dexamethasone) and osteogenic differentiation of BM-MSCs was induced by culturing cells in osteogenic medium (OM; 10% FBS, 0.1 μM dexamethasone, 10 mM β-glycerophosphate, and 0.2 mM ascorbic acid in α-MEM) were added to the confluent layer of BM-MSCs for 21 days, respectively. The adipogenic cells were stained with a working solution of oil red O for 10–15 min at room temperature. Meanwhile, the degree of extracellular matrix calcification was estimated using an alizarin red S stain.

### Hypoxic preconditioning and ischemic injury

Ischemic injury for the BM-MSCs was stimulated with hypoxia/serum deprivation (H/SD) injury as described previously [17]. Briefly, after being replaced in glucose-free DMEM without FBS, BM-MSCs were exposed to hypoxia (94% N_2_/5% CO_2_/1% O_2_) in an anaerobic system (Thermo Forma) at 37 °C for 24 h. Meanwhile, BM-MSCs incubated under normoxic conditions (37 °C in 95% air, 5% CO_2_) with full medium for equivalent periods were used as a control. Moreover, BM-MSCs were subjected to an atmosphere of 1% O_2_ and 5% CO_2_, while kept in full medium for HPC.

### Autophagy inhibition and autophagy evaluation

To study the effect of autophagy on the hypoxia injury of BM-MSCs, 3-methyladenine (3MA; 5 mM, Sigma) was administrated for 1 h to inhibit autophagy. Consistent with 3-MA, *Atg 7* gene silencing by small interfering RNAs (siRNAs) further suppressed the formation of autophagic vacuoles as described previously [16]. Briefly, BM-MSCs were plated on to six-well plates (1 × 10^5^ cells per cm^2^) for 24 h before transfection. Cells were transiently transfected with siRNAs targeting Atg7 or control siRNAs (Cell signal) using Lipofectamine™ 2000 according to the manufacturer’s protocol. All of these treatments were performed in duplicate.

The autophagy of BM-MSCs was measured by green fluorescence protein (GFP)-LC3 fusion protein, a widely accepted marker to visualize formation of autophagic vacuoles. Briefly, Lipofectamine LTX and plasmid DNA were administrated to the culture system for 4 h at 37 °C. GFP-LC3 puncta in BM-MSCs were quantified by fluorescence microscopy after different treatments. Five random fields were counted and the percentages of cells with GFP-LC3 punctate were calculated. Meanwhile, the expressions of LC-3, Beclin-1, and P62 were assessed by Western blot assay. Furthermore, autophagosomes in BM-MSCs were detected by transmission electron microscopy. Briefly, after washing with phosphate-buffered saline (PBS) and dehydration through graded ethanol, cells were embedded in epoxy resin. Ultrathin sections were prepared and stained with uranyl acetate (1%) and lead citrate (0.2%). Images were recorded using a transmission electron microscope (JEM1230; JEOL). The average numbers of the autophagic structures in the cytoplasm were calculated.

### Cell viability assay

The cell viability of BM-MSCs was assessed by 3-(4,5-dimethylthiazol-2-yl)-2,5- diphenyltetrazolium bromide (MTT) assay as described previously [17]. Briefly, BM-MSCs were plated in 96-well plates at 1 × 10^5^ cells/well. After different treatments, BM-MSCs were incubated with MTT solution (5 g/L, Sigma) at 37 °C for 4 h. The medium was then removed and 200 μL dimethyl sulphoxide (DMSO) was added to each well. The absorbance was determined at a wavelength of 490 nm. Optical density (OD) values for each group were detected in six duplicate wells and their averages were calculated. Furthermore, we also assessed cell viability with bioluminescence imaging (BLI) using the IVIS Kinetic system (Caliper, Hopkinton, MA, USA) [7]. Briefly, MSCs were plated in 24-well plates (5 × 10^4^ per well). After different treatments, cell culture media were removed. Cells were incubated with D-Luciferin reporter probe (4.5 μg/mL) and then measured using the IVIS Xenogen Kinetic system (Caliper Life Sciences, USA), using the following imaging parameters: binning, 4; F/stop 1; time, 1 min. Bioluminescent signals were analyzed using Living Image 4.0 software (Caliper, MA, USA) and quantified as photons per second per centimeter square per steridian (photons/s/cm^2^/sr).

### Measurement of VEGF, bFGF, IGF-1, and HGF

The concentrations of vascular endothelial growth factor (VEGF), basic fibroblast growth factor (bFGF), insulin-like growth factor (IGF)-1, and hepatocyte growth factor (HGF) secreted by MSCs were determined by enzyme-linked immunosorbent assay (ELISA) according to the manufacturer’s instructions. All samples and standards were measured in duplicate. In addition, paracrine secretion with and without autophagy inhibition and different HPC protocols were also evaluated.

### Apoptosis measurement

MSC apoptosis was determined by terminal deoxynucleotidyl transferase-mediated dUTP nick end-labeling (TUNEL) assay using an assay kit (In Situ Cell Death Detection Kit; Roche Diagnostics) according to the manufacturer’s instructions [7]. Briefly, after different treatments, BM-MSCs were incubated with TdT and fluorescein-labeled dUTP for 45 min at 37 °C followed by 4,6-diamidino-2-phenylindole (DAPI) for the identification of nuclei. Photographs were taken using a confocal microscope (Olympus Fluoview 2000). For each group, five random fields were counted to calculate the percentage of apoptotic cells. Meanwhile, caspase-3 activity was measured using a Caspase-3 Assay kit (Clontech, Mountain View, CA, USA) according to the manufacturer’s instructions. All of these assays were performed in a blinded manner.

### Western blot assay

Western blotting was performed following a standard protocol. Equal amounts of protein (50 μg/lane) were separated by electrophoresis on 12% SDS-PAGE gels in a Tris/HCl buffer system for 90 min at 120 V, and sequentially electrophoretically transferred to nitrocellulose (NC) membranes. After blocking with 5% nonfat dry milk (BD Biosciences) at room temperature for 1 h, membranes were subjected to immunoblotting with primary antibodies overnight at 4 °C. After incubation with the appropriate secondary antibody conjugated with horseradish peroxidase, blot bands were visualized with an enhanced chemiluminescence system (Amersham Bioscience). Densitometric analysis of Western blots was performed using VisionWorks LS, version 6.7.1. The following primary antibodies were used: rabbit anti-mice LC-3 (1:500, Cell Signaling Technology), rabbit anti-mice P62 (1:500, Cell Signaling Technology), rabbit anti-mice Beclin-1 (1:500, Cell Signaling Technology), and rabbit anti-mice β-actin (1:2000, Abcam).

### Myocardial infarction model and MSC transplantation

MI was induced in adult C57BL/6 mice by permanent ligation of the left anterior descending (LAD) artery as described previously [7, 18]. In brief, mice were anesthetized with isoflurane and mechanically ventilated. Left thoracotomy was performed and the pericardium was opened. The LAD artery was permanently ligated with a 6-0 suture. The ligation was deemed successful when the anterior wall of the left ventricle (LV) turned pale and characteristic electrocardiographic (ECG) changes were seen. Sham-operated control mice underwent the same surgical procedures except that the suture placed under the left coronary artery was not tied.

BM-MSC transplantation was performed immediately after MI. After HPC for 24 h and 48 h, respectively, the cells were collected and randomly divided into the different groups separately. The suspended BM-MSCs (1 × 10^6^) were injected directly into the peri-infarcted areas (multiple injections within the presumed infarct and the border zone) using a Hamilton syringe with a 29-gauge needle (in 20 mice in every group).

### In vivo BLI of transplanted MSCs

BLI was performed to track engrafted MSCs using an IVIS Kinetic system (Caliper, Hopkinton, MA, USA) as described previously [18]. After intraperitoneal injection with D-luciferin (375 mg/kg body weight), recipient mice were anesthetized with isoflurane and imaged for 10 min on days 1, 3, 5, and 7, and weekly until sacrificed. Peak signals (photons/s/cm^2^/sr) from a fixed region of interest (ROI) were analyzed using Living Image® 4.0 software (Caliper, MA, USA).

### Postmortem histological assays

To evaluate the survival of MSCs in ischemic myocardium, five mice were sacrificed 2 weeks after MSC transplantation. The hearts were harvested and rapidly (within a minute) fixed in 4% paraformaldehyde. Serial sections were prepared at 5 μm thickness and stained with an FITC-labeled anti-eGFP antibody and DAPI. Meanwhile, cardiomyocytes were stained with an anti-cTn I antibody. Cell engraftment was confirmed by identification of GFP expression under fluorescent microscopy. The numbers of GFP-positive cells and DAPI in each slide were calculated. The data were expressed as the percentage of GFP+/DAPI in five slides obtained from five frozen sections. To validate the proliferation of engrafted MSCs, immunofluorescence staining for the cell cycle-associated nuclear protein Ki67 was performed. The numbers of GFP and ki67 double-positive cells were calculated. Furthermore, the apoptosis of engrafted MSCs and myocardial cells were determined by TUNEL assay as previously described. TUNEL staining was performed with fluorescein-dUTP (In Situ Cell Death Detection Kit; Roche Diagnostics) for apoptotic cell nuclei and DAPI (Sigma) to stain all cell nuclei. Additional staining for troponin I or GFP was performed for the identification of myocardium and transplanted BM-MSCs, respectively. The numbers of GFP and TUNEL double-positive cells were calculated. In addition, the percentage of apoptotic cardiomyocytes was termed the apoptotic index. All of these assays were performed in a blinded manner.

### Evaluation of fibrosis

Masson’s trichrome staining was performed to assess the infarct size. Recipient mice were sacrificed for histological assay at 4 weeks after MI and MSC transplantation [19]. Hearts were fixed in 4% paraformaldehyde and embedded in paraffin. Serial sections were prepared at 5 μm thickness. Masson's trichrome stain was used to detect fibrosis in the cardiac muscle. Ten anterolateral sections from each heart were evaluated in their entirety and quantified. The fibrosis area was determined by measuring the collagen area as a proportion of the total area using Imaging Pro Plus software.

### Echocardiographic measurements

Cardiac function was measured 7 days after MI by transthoracic echocardiography at 2 days, 7 days, and weekly until sacrifice at 4 weeks post-operation using a 30-MHz transducer on a Vevo® 2100 ultrasound system (VisualSonics, CA, USA) [18, 19]. Briefly, the mice were anesthetized (2% isoflurane and oxygen) and put in a supine position. Both two-dimensional and M-mode images were recorded by a blinded investigator. The left ventricular end-systolic volume (LVESV) and diameter (LVESD) and left ventricular end-diastolic volume (LVEDV) and diameter (LVEDD) were measured to calculate left ventricular ejection fraction (LVEF) and fractional shortening (FS) via the following equations: LVEF = (LVEDV – LVESV)/LVEDV × 100%, LVFS = (LVEDD –LVESD)/LVEDD × 100%.

### Statistical analysis

The results are presented as means ± SEM. Statistics were calculated using Prism 5.0 (GraphPad Software Inc, San Diego, CA, USA). Linear regression analysis was performed to determine the correlation between two variables. Statistical comparisons for different groups were performed using either the Student’s *t* test or one-way analysis of variance (ANOVA). *p* values <0.05 were considered statistically significant.

## Results

### Characterization of BM-MSC^Fluc+GFP+^

**Fig. 1:**
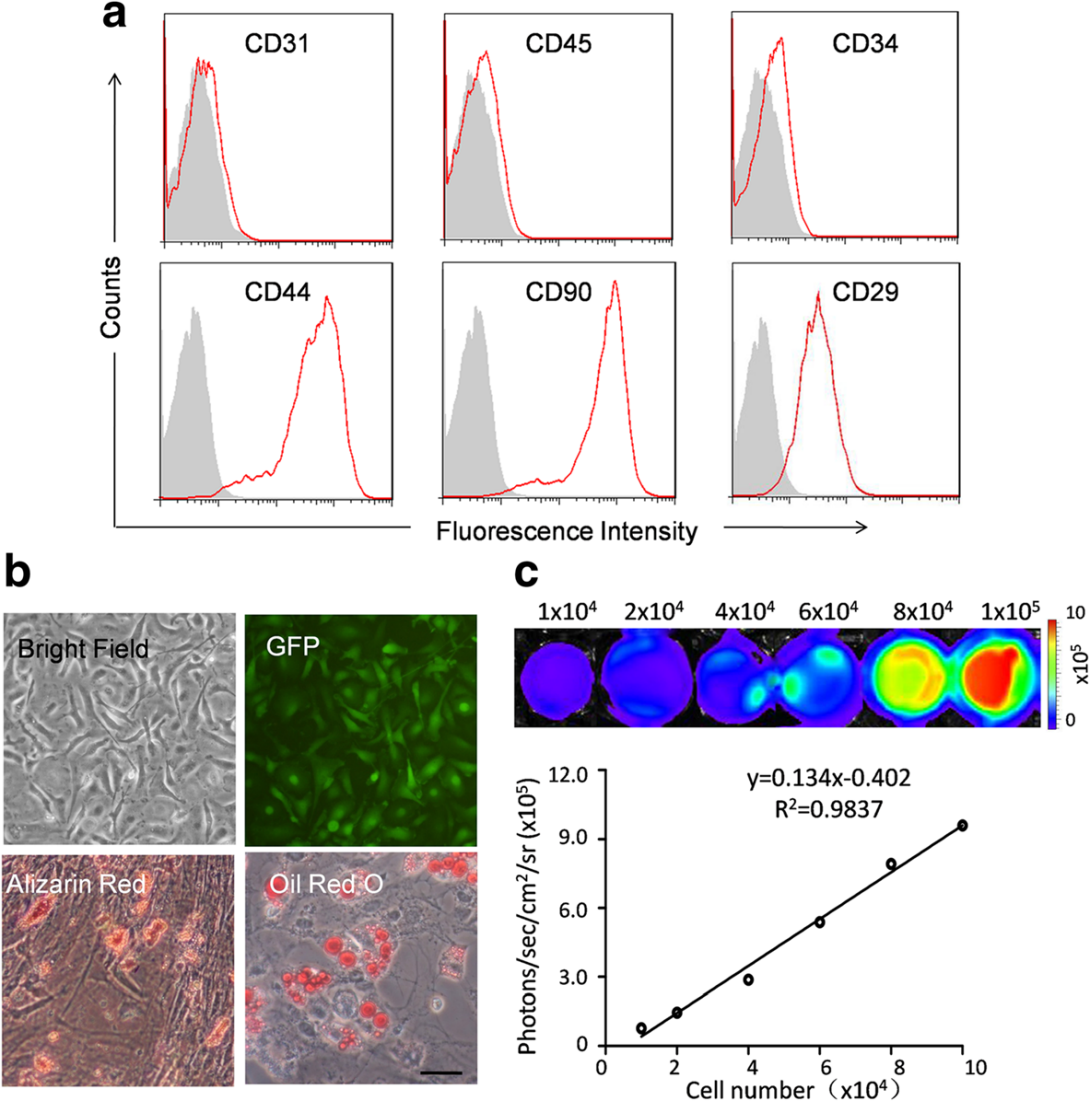
Characterization of BM-MSC ^Fluc+GFP+^. **a** Flow cytometry results show that BM-MSC^Fluc+GFP+^ were uniformly negative for CD31, CD34 and CD45, and positive for CD44, CD﻿2﻿9 and CD90. **b** The differentiation potential of BM-MSC ^Fluc+GFP+^. Fibroblast-like shaped BM-MSC^Fluc+GFP+^ were green fluorescent protein (*GFP*) positive. Adipogenesis of BM-MSC ^Fluc+GFP+^ under the adipogenic differentiation conditions was detected by oil red O staining. Osteogenesis was evaluated by alizarin red S staining (*scale bar* = 100 μm). **c** Ex vivo BLI shows a linear relationship between cell number and Fluc reporter gene activity

BM-MSCs were characterized by in vitro multilineage differentiation and flow cytometry analysis. Flow cytometry results revealed that BM-MSC^Fluc+GFP+^ were uniformly positive for the MSC markers CD44, CD90, and CD29, and negative for CD31, CD45, and CD34 (Fig. 1a). As shown in the representative images in Fig. 1b, 70% of BM-MSC^Fluc+GFP+^ had an adipocyte phenotype after adipogenic medium incubation for 21 days, which was assessed by oil red O staining. Meanwhile, alizarin red S staining for calcium deposition demonstrated that BM-MSC^Fluc+GFP+^ could also differentiate into osteogenic cells. These multilineage differentiation results revealed the pluripotency of BM-MSC^Fluc+GFP+^.

BM-MSC^Fluc+GFP+^, isolated from *Tg(Fluc-egfp)* reporter transgenic mice, constitutively express both Fluc and eGFP. As shown in the representative images in Fig. 1b, BM-MSC^Fluc+GFP+^ exhibited fibroblast-like morphology and expressed GFP. Furthermore, in vitro BLI demonstrated a robust linear correlation between the number of BM-MSC^Fluc+GFP+^ and average Fluc radiance (*r* ^2^ = 0.98; Fig .1c), indicating that BLI of Fluc was could be reliably used to monitor the viability of engrafted MSC^Fluc+GFP+^ quantitatively in vivo.

### Hypoxic preconditioning for 24 h increases the viability and growth factor secretions of BM-MSC^Fluc+GFP+^

**Fig. 2:**
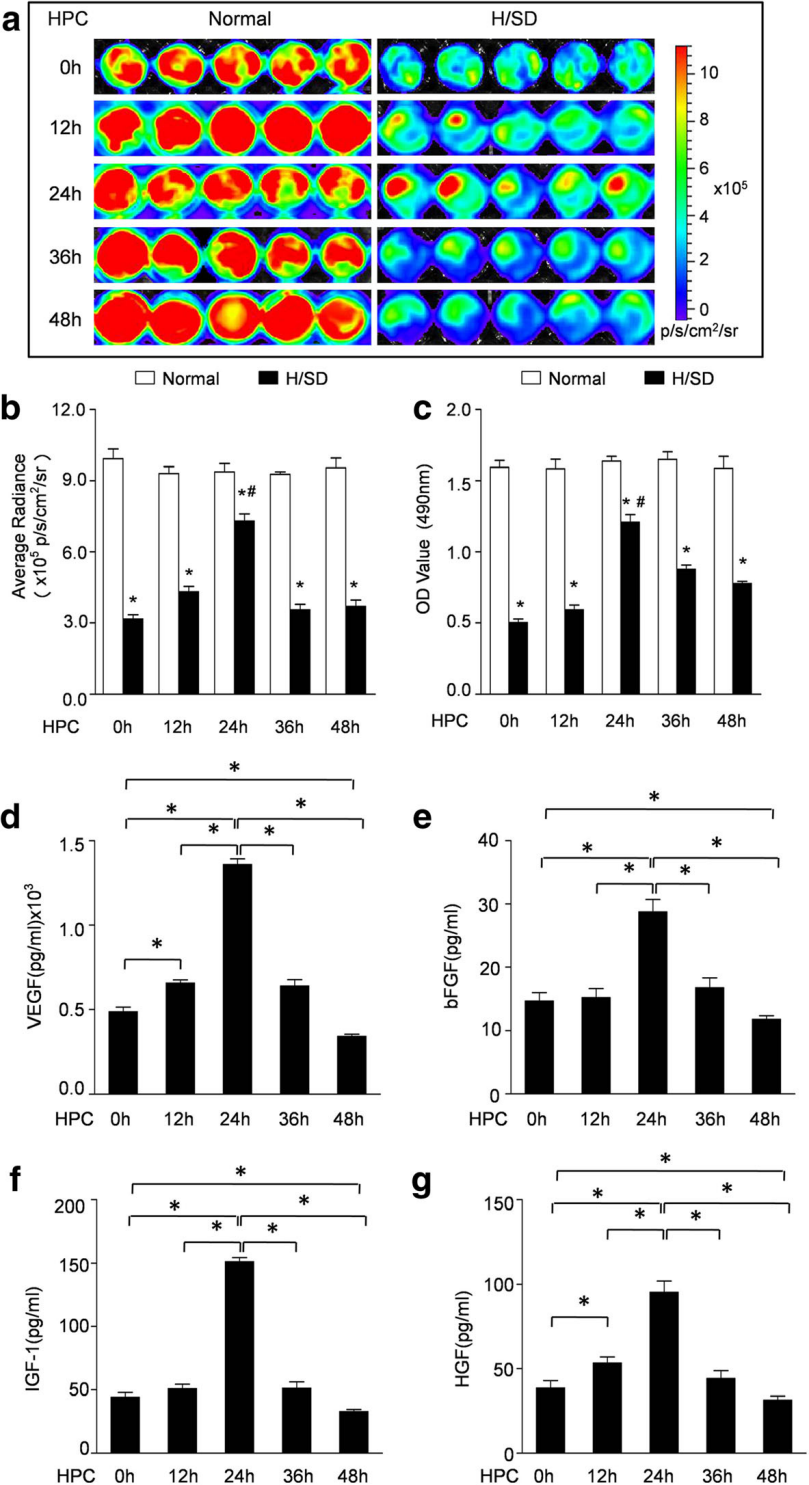
Effect of hypoxic preconditioning (*HPC*) on the viability and paracrine mechanism of BM-MSC ^Fluc+GFP+^. **a** Representative in vitro BLI results of BM-MSC^Fluc+GFP+^ with HPC for 0 h, 12 h, 24 h, 36 h, and 48 h under normal conditions and after hypoxia/serum deprivation (*H/SD*). **b** The quantification of BLI assays. **c** MTT assay demonstrated the effects of HPC (0 h, 12 h, 24 h, 36 h, and 48 h) on viability of BM-MSC^Fluc+GFP+^. **d** ELISA assay demonstrated the levels of vascular endothelial growth factor (*VEGF*) (**d**), basic fibroblast growth factor (*bFGF*) (**e**), insulin-like growth factor-1 (*IGF-1*) (**f**), and hepatocyte growth factor (*HGF*) (**g**) within BM-MSC^Fluc+GFP+^ supernatants. Data are expressed as the mean ± SEM; *n* = 5; *p < 0.05

H/SD treatment was performed to imitate ischemic conditions in vitro. In order to develop the optimal HPC protocol, MSC^Fluc+GFP+^ were preconditioned by hypoxic incubation (1% O_2_) in full medium for 12, 24, 36, or 48 h prior to H/SD injury. BLI results displayed a remarkable decline of BLI signal intensity in MSC^Fluc+GFP+^ after H/SD injury compared with normal control (3.07 ± 0.67 × 10^5^ p/s/cm^2^/sr after H/SD versus 10.48 ± 0.82 × 10^5^ p/s/cm^2^/sr under normal conditions; *p* < 0.05) (Fig. 2a and b). Moreover, the impaired viability of MSC^Fluc+GFP+^ after H/SD injury was ameliorated by HPC for 24 h (8.18 ± 0.53 × 10^5^ p/s/cm^2^/sr versus 3.07 ± 0.67 × 10^5^ p/s/cm^2^/sr for H/SD; *p* < 0.05). Meanwhile, HPC for 12 h seemed to enhance the viability of BM-MSCs compared with the H/SD group, but without statistical significance (3.87 ± 0.47 × 10^5^ p/s/cm^2^/sr versus 3.07 ± 0.67 × 10^5^ p/s/cm^2^/sr for H/SD; *p* > 0.05). However, HPC for 36 h and 48 h had no protective effect on the viability of BM-MSCs subjected to H/SD injury. In addition, MTT results also confirmed that HPC for 24 h protected the impaired viability of MSC^Fluc+GFP+^ after H/SD injury (1.23 ± 0.04 versus 0.48 ± 0.02 for H/SD; *p* < 0.05; Fig. 2c). However, there was no effect of HPC for 12, 36, or 48 h on the viability of BM-MSCs after H/SD injury (*p* > 0.05; Fig. 2c).

It has been shown that MSCs contribute to cardiac repair and regeneration at least in part by a paracrine mechanism. Therefore, we evaluated the effect of HPC on cytokine secretion in MSC^Fluc+GFP+^ by ELISA assays. As shown in Fig. 2d–g, HPC for 24 h significantly increased VEGF, bFGF, IGF-1, and HGF secreted by MSCs. However, these paracrine secretion levels were decreased after administration of 3-MA and Atg7 siRNA (Additional file 1: Figure S1). Furthermore, HPC for 48 h had a downregulation effect on VEGF, bFGF, IGF-1, and HGF secreted by MSCs. However, those paracrine secretion levels were dramatically elevated after administration of 3-MA and Atg7 siRNA (Additional file 2: Figure S2).

### The apoptosis of BM-MSC^Fluc+GFP+^ was decreased by hypoxic preconditioning for 24 h while increased by hypoxic preconditioning for 48 h

**Fig. 3:**
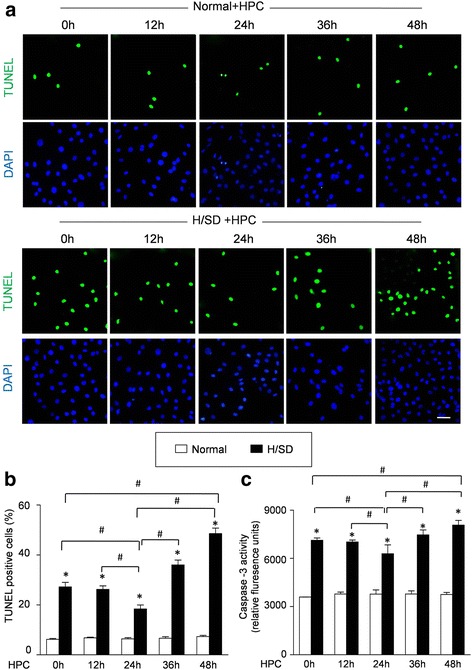
Effect of hypoxic preconditioning (*HPC*) on the apoptosis of BM-MSCs. **a** Representative immunofluorescence images of terminal deoxynucleotidyl transferase-mediated nick-end labeling (*TUNEL*) (*green* fluorescence) and 4,6-diamidino-2-phenylindole (*DAPI*) (*blue* fluorescence) in BM-MSCs under normal conditions and after hypoxia/serum deprivation (*H/SD*) (*Scale bars* = 20 μm). **b** Quantification of the apoptotic BM-MSCs was presented as the percentage of apoptotic cells. **c** Histogram illustrated the caspase-3 enzymatic activity in BM-MSCs in all groups. Data are expressed as means ± SEM; *n* = 5; *p < 0.05H/SD vs. Normal,﻿^ #^ *p*﻿<﻿0.0﻿5﻿

To analyze the anti-apoptotic effect of HPC, TUNEL assay and caspase-3 activity assay were performed to evaluate the apoptosis of BM-MSCs induced by H/SD. Representative immunofluorescence images and quantitative analyses are shown in Fig. 3a and b. H/SD injury increased the TUNEL-positive cells (27. 32 ± 1.41% versus 5.63 ± 0.72% for normal; *p* < 0.05), which was decreased by HPC for 24 h (14. 69 ± 1.13% versus 27. 32 ± 1.41% for H/SD; *p* < 0.05). Moreover, HPC for 12 h had no effect on the apoptosis of BM-MSCs induced by H/SD injury (*p* > 0.05). Interestingly, the percentage of TUNEL-positive cells in the HPC 36 h group was 36.15 ± 1.57%, higher than that in the H/SD group without statistical significance (*p* > 0.05). Furthermore, HPC for 48 h significantly increased the apoptosis of BM-MSCs compared with the H/SD group (44. 73 ± 1.63% versus 27. 32 ± 1.41% for H/SD; *p* < 0.05), which was reduced by 3-MA and Atg7 siRNA (*p* < 0.05; Additional file 3: Figure S3). Concurrently, we also found that the caspase-3 enzymatic activity in the H/SD group was significantly increased compared with that in the normal group (7.13 ± 0.19 × 10^3^ RFU versus 3.58 ± 0.02 × 10^3^ RFU for normal; *p <* 0*.*05) (Fig. 3c). Furthermore, HPC for 24 h decreased the enhanced caspase-3 enzymatic activity induced by H/SD (5.72 ± 0.52 × 10^3^ RFU versus 7.13 ± 0.19 × 10^3^ RFU for H/SD; *p <* 0*.*05). However, HPC for 48 h significantly increased the caspase-3 enzymatic activities compared with the H/SD group (8.22 ± 0.38 × 10^3^ RFU versus 7.13 ± 0.19 × 10^3^ RFU for H/SD; *p <* 0*.*05). Taken together, these data suggested that HPC for 24 h prevented apoptosis of BM-MSCs while HPC for 48 h promoted apoptosis of BM-MSCs induced by H/SD injury.

### Hypoxic preconditioning increases autophagy in BM-MSCs

**Fig. 4:**
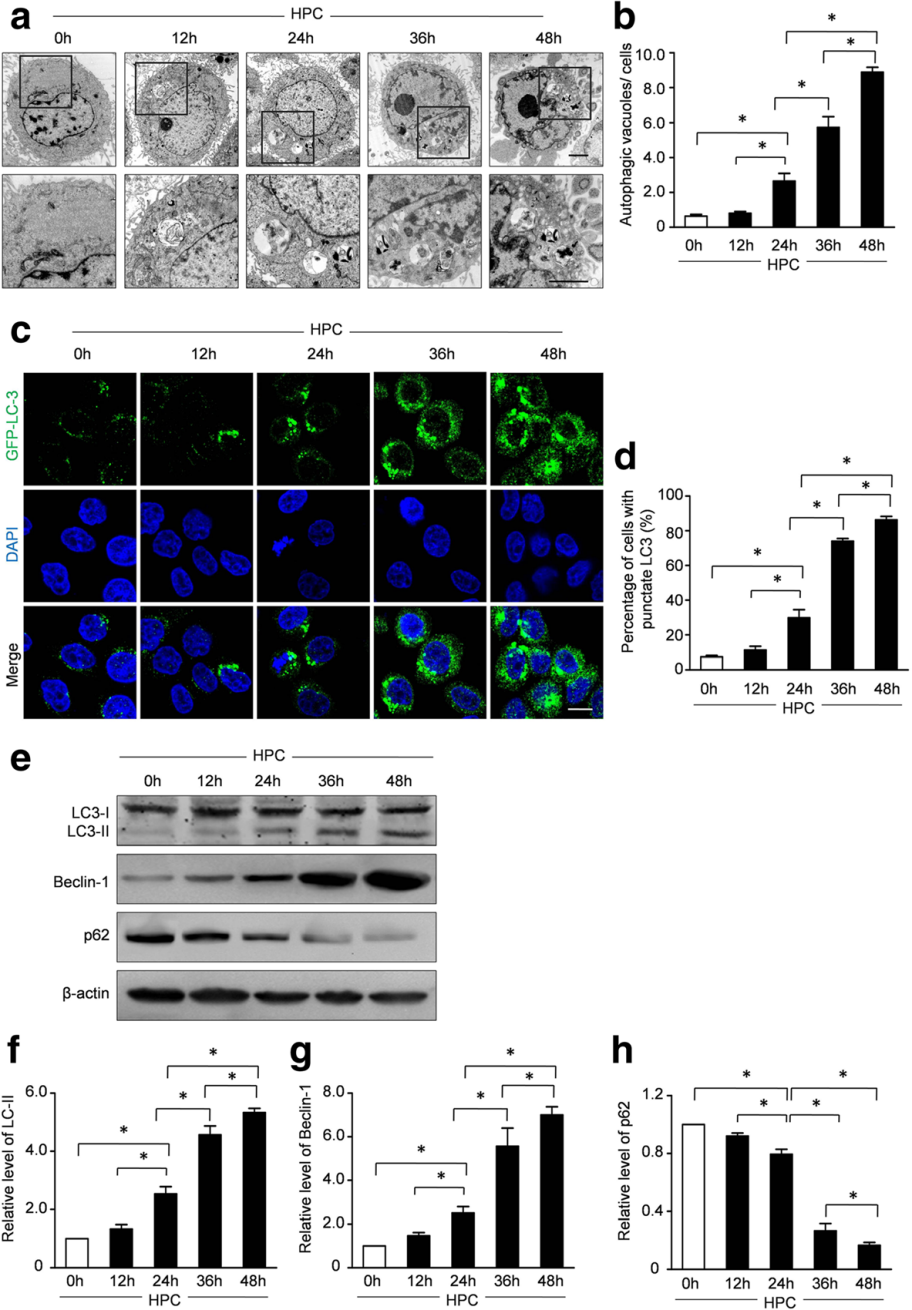
Effect of hypoxic preconditioning (*HPC*) on the autophagy of BM-MSCs. **a** Representative electron micrographs revealed the autophagic vacuole formation in BM-MSCs. **b** Quantification of the average numbers of the autophagic structures in the cytoplasm. **c** Autophagy flux was analyzed in BM-MSCs infected with Ad-GFP-LC3 adenovirus. Representative immunofluorescence images of green fluorescent protein (*GFP*)-LC3 (*green* fluorescence) and 4,6-diamidino-2-phenylindole (*DAPI*) (*blue* fluorescence) in BM-MSCs with HPC for the indicated time points. *Scale bars* = 20 μm. **d** Quantification of autophagy flux was presented as the percentage of BM-MSCs with punctate LC3 in all groups. **e** Representative Western blots of LC3-I/LC3-II, Beclin-1, and P62 in BM-MSCs with HPC for 0 h, 12 h, 24 h, 36 h, and 48 h, respectively. Semiquantification of the protein expressions of LC3-II (**f**), Beclin-1 (**g**), and p62 (**h**) at the indicated time points. Data are expressed as means ± SEM; *n* = 5; *p < 0.05

To explore the autophagy level under hypoxic conditions, BM-MSCs were transfected with GFP-LC3 to demonstrate the LC3 expression. Meanwhile, the protein expressions of LC3-I/LC3-II, P62, and Beclin-1 were also assessed by Western blot assay. Furthermore, the autophagosomes in BM-MSCs were also detected by transmission electron microscopy. Autophagosome formation was increased by hypoxic preconditioning time-dependently (Fig. 4a). Moreover, the representative immunofluorescence microphotographs and the quantitative analyses in Fig. 4c and d revealed that the percentage of cells with punctate LC3 was 5.12 ± 0.07% under normal conditions. Conversely, the percentages of cells with punctate LC3 were increased by HPC time-dependently. Furthermore, the representative Western blot assay and semiquantitative analysis revealed that HPC significantly increased the expressions of LC3-II and Beclin-1 in BM-MSCs. However, the levels of expression of p62 were decreased by HPC (Fig. 4e–h). Taken together, these results suggested that HPC activated autophagy in BM-MSCs.

### Autophagy inhibition by 3-methyladenine and Atg7 siRNA

To determine the effect of autophagy on the protective effect of HPC, autophagy in BM-MSCs was inhibited by the autophagy inhibitor 3-MA and Atg7 siRNA, respectively. The increased punctate LC3 formation induced by HPC for 24 h was blocked by 3-MA and Atg7 siRNA (Additional file 4: Figure S4). Furthermore, quantitative analyses revealed that the percentage of BM-MSCs with punctate LC3 in the 24-h HPC group was 29.31 ± 4.72%, significantly higher than that under normal condition (6.25 ± 1.14%; *p* < 0.05). Conversely, the percentage of BM-MSCs with punctate LC3 in the HPC + 3-MA group and HPC + Atg7 siRNA group was 12.32 ± 3.72% and 9.15 ± 0.79%, respectively, significantly less than that in the HPC group (*p* < 0.05). Collectively, these results indicated that 3-MA and Atg7 siRNA were reliably able to inhibit autophagy in BM-MSCs.

### Autophagy contributes to the protective effect of HPC against hypoxic stress

**Fig. 5:**
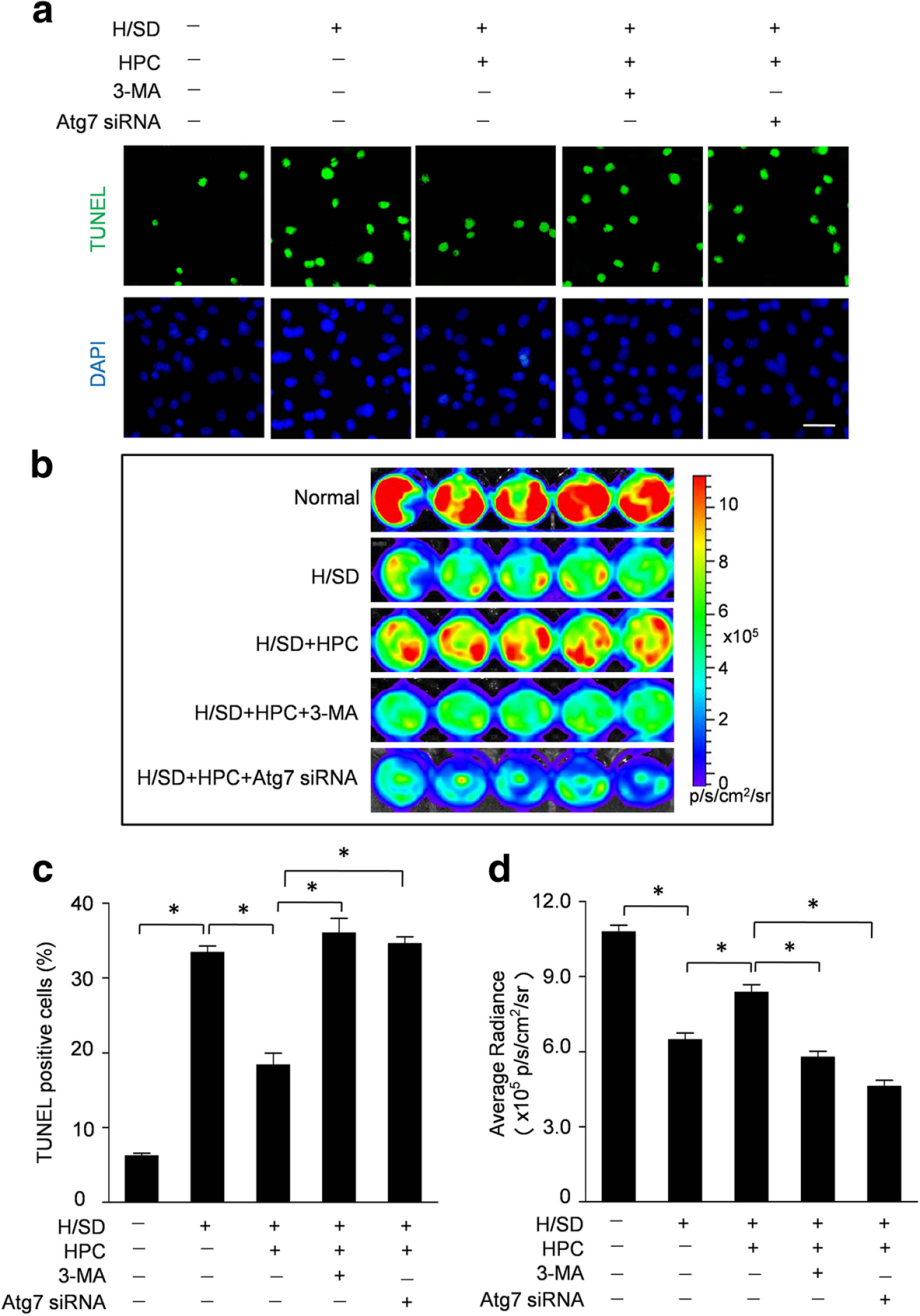
Autophagy modulated the protective effect of hypoxic preconditioning (*HPC*) on BM-MSCs. **a** Representative terminal deoxynucleotidyl transferase-mediated nick-end labeling (*TUNEL*) images of BM-MSCs treated by HPC for 24 h with or without autophagy inhibition by 3-methyladenine (*3-MA*) and Atg7 small interfering RNA (*siRNA*) (*Scale bars* = 20 μm). **b** Representative in vitro BLI results of BM-MSCs^Fluc+GFP+^ in all groups. **c** The quantification of the apoptotic BM-MSCs. **d** The quantification of the in vitro BLI assays. Data are expressed as means ± SEM; *n* = 5; *p < 0.05. *DAPI* 4,6-diamidino-2-phenylindole, *H/SD* hypoxia/serum deprivation

To gain an insight into the role of autophagy on the protective effect of HPC against H/SD injury, we inhibited the autophagy in BM-MSCs by Atg7 siRNA and 3-MA. Representative immunofluorescence images from the TUNEL assay (Fig. 5a) revealed that HPC for 24 h significantly decreased the apoptosis in BM-MSCs compared with the H/SD group, which was abolished by 3-MA treatment and Atg7 siRNA. Furthermore, quantitative analyses (Fig. 5c) revealed that the percentage of TUNEL-positive cells in the hypoxia group was 33.73 ± 2.25%, significantly higher than that in the normal group (5.76 ± 1.12%; *p* < 0.05) and the HPC + H/SD group (18.35 ± 2.54%; *p* < 0.05). However, the percentages of TUNEL-positive BM-MSCs in the H/SD + HPC + 3-MA group and the H/SD + HPC + Atg7 siRNA group were 35.14 ± 3.19% and 32.31 ± 2.42%, respectively, significantly higher than that in the HPC + H/SD group (18.35 ± 2.54%; *p* < 0.05). Concurrently, BLI assays were performed to evaluate the viability of BM-MSCs (Fig. 5b). Moreover, the quantitative analyses (Fig. 5d) indicated that the impaired viability of MSCs after H/SD injury was ameliorated by HPC for 24 h (8.23 ± 0.46 × 10^5^ p/s/cm^2^/sr vs. 6.05 ± 0.38 × 0^5^ p/s/cm^2^/sr for H/SD; *p* < 0.05). However, autophagy inhibition with 3-MA and Atg7 siRNA further abrogated HPC-induced preservation of the viability of BM-MSCs as manifested by the BLI signal (5.89 ± 0.43 × 10^5^ p/s/cm^2^/sr for HPC + H/SD + 3-MA, 5.12 ± 0.53 × 10^5^ p/s/cm^2^/sr for HPC + H/SD + Atg7 siRNA versus 8.23 ± 0.46 × 10^5^ p/s/cm^2^/sr for H/SD + HPC; *p* < 0.05).

### HPC for 24 h promotes survival of engrafted BM-MSCs

**Fig. 6:**
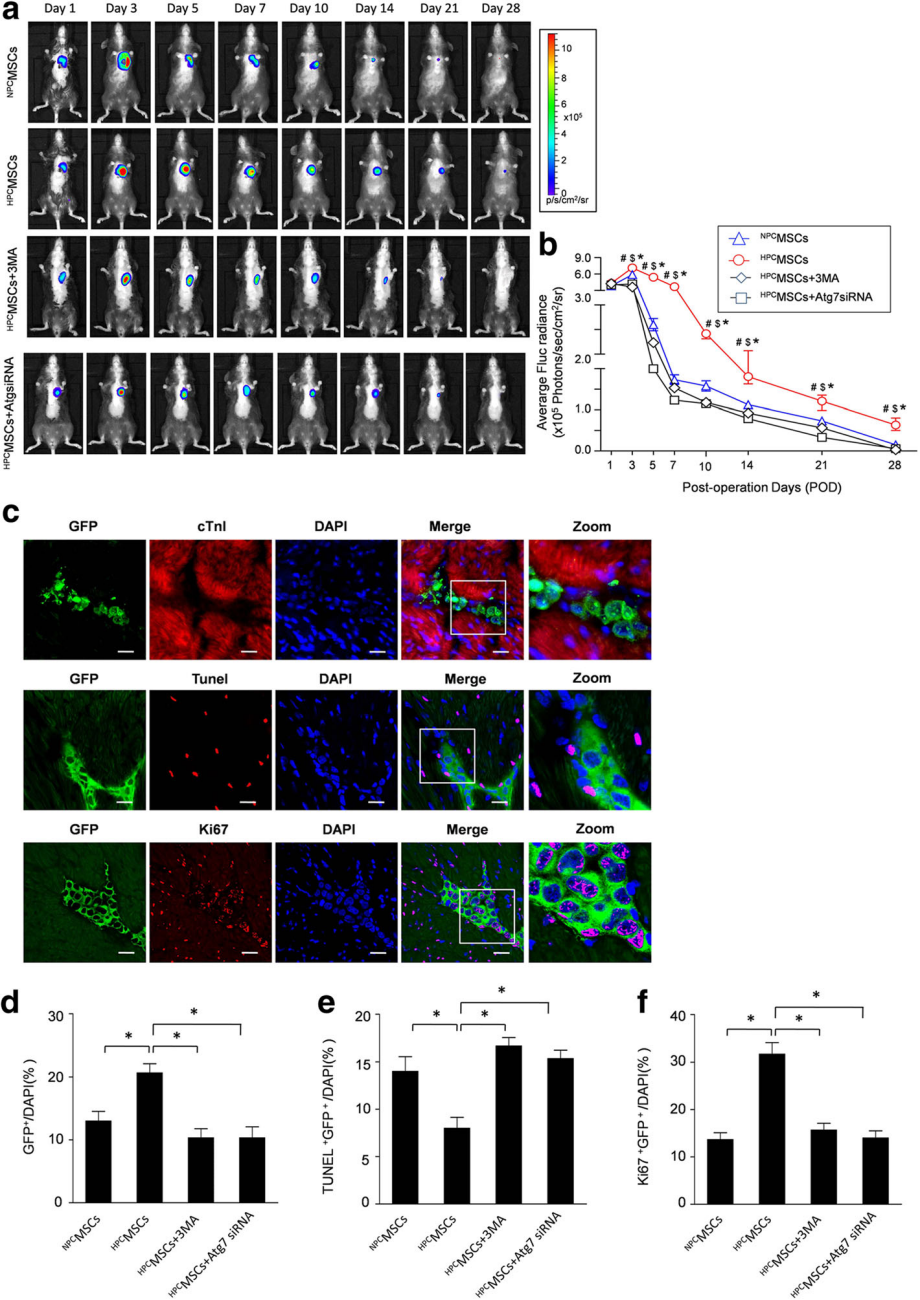
Evaluation of the survival of transplanted BM-MSC^Fluc+GFP+^. **a** Representative longitudinal BLI spatiotemporally tracked BM-MSC ^Fluc+GFP+^ survival in normal preconditioned mesenchymal stem cells (^*NPC*^ *MSCs*) (*top row*, *n* = 10), hypoxic preconditioned MSCs (^*HPC*^ *MSCs*) (*second row*, *n* = 10), the ^HPC^MSCs + 3-methyladenine (*3-MA*) group (*third row*, *n* = 10), and the ^HPC^MSCs + Atg7 small interfering RNA (*siRNA*) group (*bottom row*, *n* = 10). Color scale bar values are in photons/s/cm^2^/sr. **b** Quantitative analysis of Firefly luciferase (*Fluc*) optical signals on fixed regions of interest (ROI). **c** Representative confocal laser microscopic images of engrafted BM-MSC^Fluc+GFP+^ (green fluorescent protein (*GFP*): *green* fluorescence), cTnI (*red, upper panel*), terminal deoxynucleotidyl transferase-mediated nick-end labeling (*TUNEL*) (*red, middle panel*), Ki67 (*red, lower panel*), and 4,6-diamidino-2-phenylindole (*DAPI*) (*blue* fluorescence) at 2 weeks after transplantation. *Scale bar* = 50 μm. Quantitative analysis of the ratio of GFP/DAPI (**d**), GFP and TUNEL double-positive cells (GFP^+^TUNEL^+^/DAPI, **e**) and GFP and Ki67 double-positive cells (GFP^+^Ki67^+^/DAPI, **f**) in all groups. Data are expressed as means ± SEM; *n* = 5; *p < 0.05

To determine the effect of HPC on the viability of BM-MSCs transplanted into infarcted hearts, BLI was performed for 4 weeks. Representative BLI results and the quantitative analyses revealed a progressive decay of the BLI signal within 4 weeks after transplantation. In contrast, HPC for 24 h facilitated the survival of engrafted BM-MSCs, which was significantly obliterated by autophagy inhibition with 3-MA and Atg7 siRNA (Fig. 6a and b). Furthermore, HPC for 24 h facilitates the survival of engrafted BM-MSCs, while HPC for 48 h showed a sharply progressive decay of the BLI signal after transplantation (Additional file 5: Figure S5).

To further confirm the in vivo BLI results for BM-MSC survival, the hearts were harvested 2 weeks after cell transplantation and stained for GFP. Cell survival was evaluated by calculating the ratio GFP/DAPI. The GFP-positive cells were more frequently observed in mice administered with HPC MSCs (^HPC^MSCs). The ratio GFP/DAPI in the ^HPC^MSC group was 21.6 ± 4.3%, significantly higher than that in the normal preconditioned MSC (^NPC^MSC) group (12.2 ± 3.5%; *p* < 0.05) (Fig. 6d). Furthermore, we also observed lower percentages of TUNEL and GFP double-positive cells (7.3 ± 4.1%) in the ^HPC^MSC group compared with the ^NPC^MSC group (13.3 ± 3.9%; *p* < 0.05) (Fig. 6e). Additionally, the proliferation of engrafted MSCs was detected by Ki-67 staining. The ratio of GFP and Ki67 double-positive cells in the ^HPC^MSC group was 30.5 ± 4.9%, significantly higher than that in the ^NPC^MSC group (12.3 ± 3.3%; *p* < 0.05) (Fig. 6f). These data suggest that HPC increases the survival of BM-MSCs in post-infarct hearts. In contrast, the beneficial effects of HPC on engrafted BM-MSCs were significantly abolished by autophagy inhibition with 3-MA and Atg7 siRNA.

### Anti-apoptotic and pro-angiogenic effects facilitated by HPC were abolished by autophagy inhibition

**Fig. 7:**
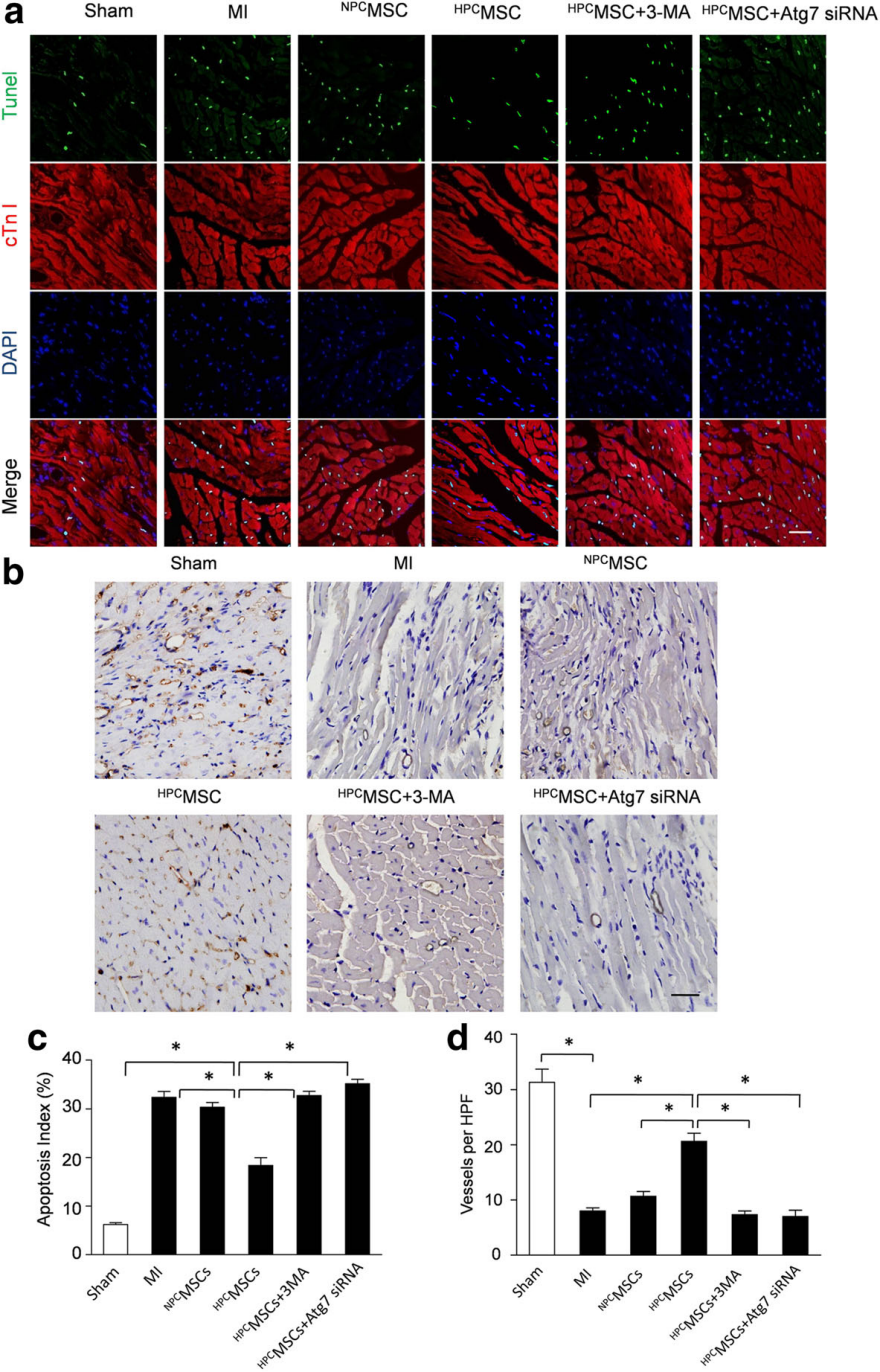
HPC facilitated the anti-apoptotic and pro-angiogenic effects of BM-MSCs. **a** Representative terminal deoxynucleotidyl transferase-mediated nick-end labeling (*TUNEL*) images for cell apoptosis in the border zone around the infarcted area in mouse hearts. Apoptotic nuclei were identified as TUNEL positive (*green* fluorescence). The myocardium was stained using a monoclonal antibody against cTnI (*red* fluorescence) and total nuclei by 4,6-diamidino-2-phenylindole (*DAPI*) counterstaining (*blue* fluorescence). *Scale bar* = 50 μm. **b** Capillaries in the infarct border zone were determined by immunohistochemical staining for CD31-positive cells in all groups. *Scale bars* = 50 μm. Quantitative analysis of apoptotic nuclei (**c**) and capillaries in the infarct border zone (**d**). Data are expressed as means ± SEM; *n* = 5; *p < 0.05. *3-MA* 3-methyladenine, ^*HPC*^ *MSCs* hypoxic preconditioned mesenchymal stem cells, *HPF* high-powered field, *MI* myocardial infarction, ^*NPC*^ *MSCs* normal preconditioned mesenchymal stem cells, *Sham* sham-operated, *siRNA* small interfering RNA

As shown by representative immunofluorescence images in Fig. 7a, apoptotic cardiomyocytes, as manifested by TUNEL positivity (in green), were more frequently observed in the MI group than in the sham-operated group (32.31 ± 2.48% versus 5.32 ± 1.03%; *p* < 0.05) (Fig. 7c). Moreover, transplantation of ^HPC^BM-MSCs significantly decreased the apoptosis of cardiomyocytes compared to the ^NPC^BM-MSC group (18.31 ± 2.75% versus 27.52 ± 1.72%; *p* < 0.05). However, the percentages of TUNEL-positive cardiomyocytes in the ^HPC^MSC + 3-MA group and the ^HPC^MSCs + Atg7siRNA group were 31.87 ± 2.04% and 34.24 ± 2.21%, respectively, significantly higher than that in the ^HPC^MSC group (18.31 ± 2.75%; *p* < 0.05).

Furthermore, we evaluated revascularization in the peri-infarct zone (Fig. 7b and d). The vessel densities, as evaluated by immunohistochemistry of CD31, were reduced in the MI group compared to the sham-operated group. Transplantation of ^HPC^BM-MSCs significantly increased the number of capillaries in CD31^+^ cells compared to the ^NPC^BM-MSCs group. However, the anti-apoptotic and the pro-angiogenic effects of ^HPC^BM-MSCs were abolished by autophagy inhibition by 3-MA and Atg7 siRNA.

### Hypoxia-preconditioned BM-MSCs reduce fibrosis and preserved heart functional recovery after MI

**Fig. 8:**
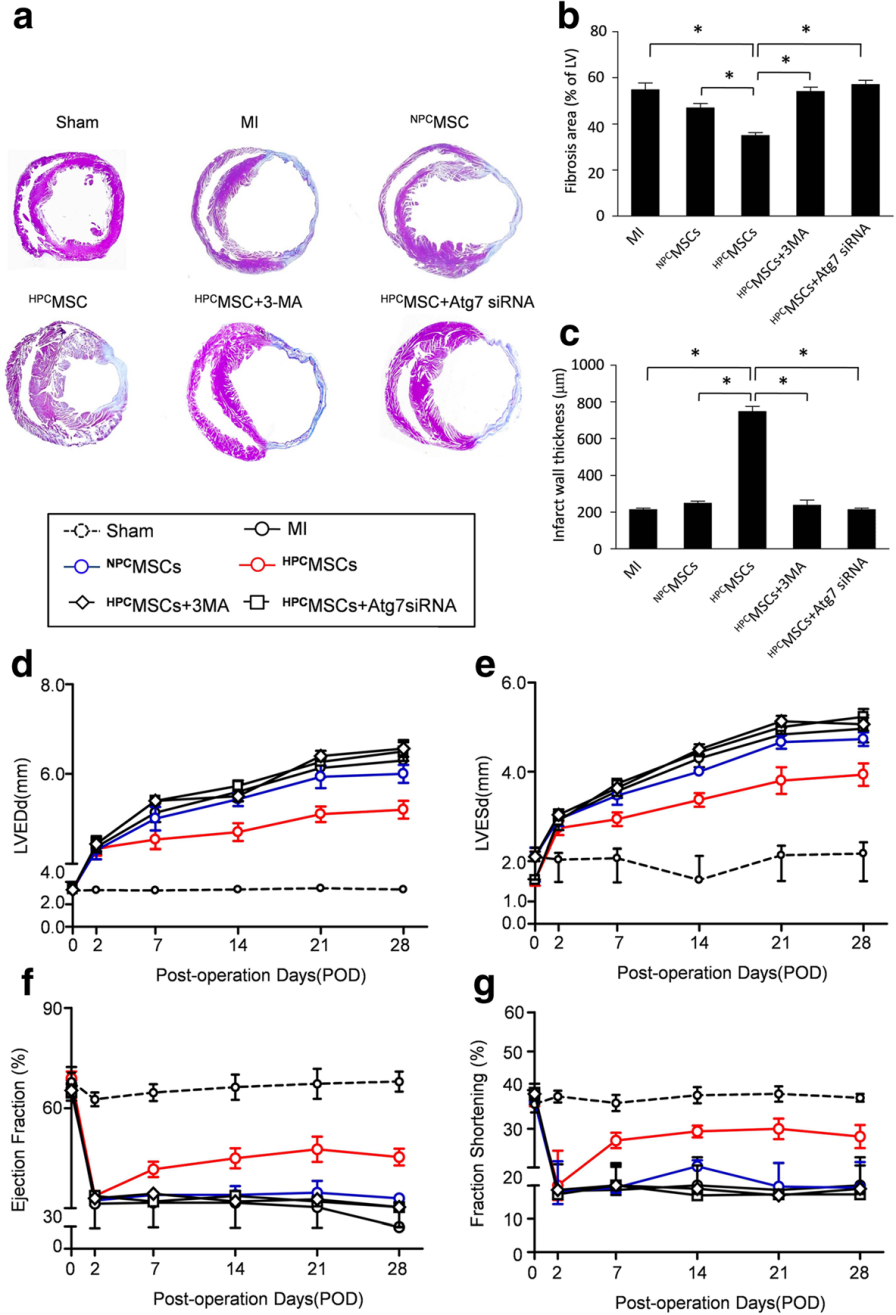
Evaluation of fibrosis and heart function after myocardial infarction (*MI*). **a** Representative Masson’s trichrome staining revealed left ventricular fibrosis 4 weeks after MI (magnification 4×). Quantitative analysis of the fibrotic area (**b**) and infarct wall thickness (**c**). Histograms illustrating heart function parameters: left ventricular end-diastolic diameter (*LVEDd*, **d**), left ventricular end-systolic diameter (*LVESd*, **e**), left ventricular ejection fraction (**f**) and left ventricular fractional shortening (**g**). Data are expressed as means ± SEM; *n* = 5; *p < 0.05. *3-MA* 3-methyladenine, ^*HPC*^ *MSCs* hypoxic preconditioned mesenchymal stem cells, *LV* left ventricle, ^*NPC*^ *MSCs* normal preconditioned mesenchymal stem cells, *Sham* sham-operated, *siRNA* small interfering RNA

To study the effects of HPC on the therapeutic efficiency of engrafted BM-MSCs, we performed fibrosis and function analysis. Representative Masson’s trichrome staining results showed that ^HPC^BM-MSCs decreased fibrosis after MI (Fig. 8a and b). Moreover, infarct wall thickness (Fig. 8c) in the hearts from mice transplanted with hypoxic preconditioned BM-MSCs was increased compared to that from mice treated with normoxia cultured BM-MSCs. Serial echocardiographic analysis indicated that the baseline parameters were similar in all groups. The LV dimensions (LVEDD and LVESD) were increased after MI. Meanwhile, the LV dimensions were decreased in the ^HPC^BM-MSC group compared with the MI and ^NPC^BM-MSC groups (Fig. 8d and e). Moreover, transplantation of ^HPC^BM-MSCs also manifested a trend towards improvement of cardiac performance over the 4 weeks after MI. However, the apparent benefit of ^HPC^BM-MSC transplantation was abolished by autophagy inhibition with 3-MA and Atg7 siRNA(Fig. 8f and g). Collectively, these results suggest that the therapeutic benefits of BM-MSC transplantation after MI is enhanced by hypoxic preconditioning via an autophagy-dependent mechanism.

## Discussion

In the present study, we demonstrate for the first time that hypoxic preconditioning exerts a protective effect on BM-MSCs against hypoxia and nutrient deprivation in vitro, associated with autophagy regulation. In addition, hypoxic preconditioning also increases the survival of BM-MSCs after transplantation and further enhances their therapeutic potential for MI in vivo. Furthermore, the beneficial effects generated from the hypoxia preconditioning of BM-MSCs are mediated in an autophagy-dependent manner. Overall, this study demonstrates that hypoxic preconditioning may be a potential optimizing target for BM-MSC-based cellular therapy for MI.

Stem cell-based therapy for MI has been considered as a potential strategy for MI. However, previous studies revealed that the therapeutic benefits seem to be relatively modest [7]. Meanwhile, our clinical trials also revealed that intracoronary administration of autologous bone marrow mononuclear cells (BMMNC) can lead to no significant myocardial functional improvement after 4 years of follow-up [20]. Poor viability and low cell retention of donor cells in the ischemic myocardium are thought to be a primary limitation for the clinical application of cell therapy. Mangi et al. [21] and van der Bogt et al. [22] found that autologous BM-MSCs had undergone acute death within 1 week after transplantation and the survival rate of engrafted cells was only 1% in the ischemic heart. Consistently, our previous studies also demonstrated a massive cell death between days 3 and 7 after transplantation [7, 18]. In the present study, we performed in vivo BLI assay to track the engrafted BM-MSCs, which were isolated from reporter transgenic mice and constitutively express Fluc and GFP [18]. The BLI results showed acute cell death within 7 days after engraftment into the infarcted myocardium. Moreover, BM-MSC transplantation could not improve the cardiac function significantly, which was consistent with previous studies [23]. Furthermore, the histological assays revealed that GFP^+^ cells were increased in the ^HPC^MSC group, indicating that HPC enhanced the survival of BM-MSCs after transplantation. In addition, the immunofluorescent staining for Ki-67 and TUNEL also revealed that HPC increased the viability of transplanted MSCs with decreased apoptosis. Taken together, these data suggested that HPC enhanced the survival and proliferation of engrafted cells, which contributed to prolonged duration of BM-MSCs in the infarcted heart.

Although the cause of transplanted cell death remains to be elucidated, the noxious milieu in the ischemic myocardium, coupled with enhanced inflammation, oxidative stress, and accumulation of cytotoxic substances, offers a significant challenge to the transplanted donor stem cells. Therefore, strategies aimed at improving the adaptation of transplanted MSCs to the harsh hypoxic conditions are crucial for improving the efficiency of cell therapy. Many methods, including gene modification [21] and pharmaceutical approaches [7, 18], have been suggested to be effective in reinforcing the viability of the donor cells. However, these approaches are not suitable for the clinic because of some potential drawbacks, including insertional mutagenesis, high manufacturing costs, and being time consuming. Therefore, simpler and safer methods to enhance the therapeutic efficacy need to be explored.

Preconditioning of donor cells has recently attracted attention as an optimized strategy to increase the therapeutic efficacy of stem cell-based treatment of ischemic diseases [24, 25]. Although MSCs normally reside in a physiologically hypoxic niche, such as bone marrow and adipose tissue, the ex vivo culture condition is normoxic [26, 27]. Once transplanted into the ischemic myocardium, MSCs encounter severe hypoxia and a cytokine-rich microenvironment, which results in extensive apoptosis. However, sublethal hypoxic culture (1%–3% O_2_) before exposure to the severe ischemia has been proved to be beneficial for MSCs, as this oxygen tension is more similar to the physiologic niche of MSCs. Previous studies demonstrated that hypoxic preconditioning increased the survival and therapeutic potency of stem cells [9, 26, 28–30]. However, the detailed preconditioning protocols used in previous studies varied widely. In the present study, we found that the beneficial effects of HPC with 1% O_2_ for 24 h are most pronounced, which was in line with most HPC protocols described for human or animal bone marrow MSCs. Long-term ischemic preconditioning, however, proved to be detrimental to BM-MSCs. Therefore, the optimized HPC protocol (with both 1% O_2_ for 24 h and 48 h) was used in our study.

Previous studies have demonstrated that HPC can increase the therapeutic effects of transplanted stem/progenitor cells in ischemic diseases, such as in the limb [31], cerebral [32], renal [33], and spinal cord [34]. To intuitively reveal the effect of HPC on the survival of BM-MSCs in the ischemic heart, we performed the in vivo BLI assay and found the ameliorated survival of ^HPC^MSCs compared with ^NPC^MSCs. Moreover, transplantation with ^HPC^MSCs was effective at preventing cardiomyocyte apoptosis, decreasing myocardial fibrosis, increasing the number of new capillaries, and improving cardiac function. Taken together, our results indicated that HPC for 24 h promoted the viability of BM-MSCs in the infarcted heart and even enhanced the therapeutic efficiency for MI, while long-term HPC promoted apoptosis and the mortality rate of BM-MSCs in the infarcted heart.

The mechanisms underlying the beneficial effects of hypoxia preconditioning on MSCs remain incompletely understood. Autophagy is a tightly regulated catabolic mechanism that is essential to maintain homeostasis and normal physiological functioning [35]. It has been proved that various stressful conditions, including hypoxia and nutrition deprivation, can induce autophagy [36]. However, the relationship between autophagy and cell death is controversial. Moreover, the detailed effect of HPC on autophagy of MSCs has not been fully understood. Therefore, we hypothesized that the protective effects of HPC on BM-MSCs are mediated by regulating autophagy. In the present study, hypoxic preconditioning (1% oxygen) caused time-dependent autophagy, which was demonstrated by an increase in LC3 expression and the formation of autophagic vacuoles (Fig. 4). However, HPC for 36 h or 48 h was detrimental to BM-MSCs in vitro, indicating that dramatically upregulated autophagy induced by long-term HPC may reversely lead to programmed cell death. Furthermore, 3-MA, an autophagic inhibitor that inhibits class III PI3K, blocked the HPC-mediated protection. Consistently, inhibition of the autophagic initiation by Atg7 siRNA also eliminated the beneficial effect of HPC. Moreover, the therapeutic effects of HPC-MSC transplantation were also abolished by autophagy inhibition with 3-MA or Atg7 siRNA. Taken together, these results confirm that modest HPC-induced protection (with 1% O_2_ for 24 h) is likely mediated by regulating the autophagic pathway.

There is still substantial controversy on the detailed mechanisms of stem cell therapy for ischemic diseases. Although transdifferentiation into cardiomyocytes in cardiac niches was proposed as the key mechanism, a growing body of evidence suggests that paracrine mechanisms mediated by MSCs may play a more essential role [37]. Our previous study revealed that BM-MSCs secreted all kinds of bioactive factors, such as VEGF, bFGF, HGF, and IGF-1. In the present study, we found that the paracrine secretion effects of BM-MSCs were enhanced by our optimized HPC protocol (with 1% O_2_ for 24 h). Moreover, VEGF, bFGF, HGF, and IGF-1 were downregulated with HPC for 48 h. However, those secretions were dramatically elevated after autophagy inhibition (by 3-MA or Atg7 siRNA administration). Therefore, appropriate autophagy induced by HPC for 24 h could significantly promote paracrine secretion. Furthermore, transplantation of ^HPC^MSCs significantly increased the number of capillaries in the peri-infarct zone, indicating that HPC enhanced the angiogenic potential of BM-MSCs. However, both apoptosis and autophagy levels were significantly increased with HPC for 48 h. Furthermore, the apoptosis was decreased, while paracrine secretions were dramatically elevated, after autophagy inhibition by 3-MA and Atg7 siRNA. Therefore, excessive autophagy can increase the apoptosis level thereby affecting BM-MSC function. Taken together, our results suggest that autophagy plays a double-edged sword under different HPC protocols.

## Conclusions

In conclusion, the current study confirmed that HPC for 24 h has the potential to enhance the functional survival of MSCs implanted into the infarcted heart. In addition, MSCs with HPC for 24 h before transplantation exhibited a significant improvement in cardiac function. The favorable effect of HPC on MSCs could be attributed to regulation of autophagy. Therefore, our results suggest that appropriate hypoxic preconditioning may be a promising novel approach for optimizing MSC transplantation therapy for MI.

## Additional files

Additional file 1: Figure S1. The effect of autophagy inhibition on the paracrine secretions of BM-MSCs treated by HPC for 24 h. ELISA assay demonstrated the levels of VEGF (A), bFGF (B), IGF-1 (C), and HGF (D) secreted by BM-MSCs treated by HPC for 24 h with and without autophagy inhibition by 3-MA and Atg7 siRNA. Data are expressed as means ± SEM; *n* = 5; *p < 0.05. (TIF 626 kb)

Additional file 2: Figure S2. Effect of HPC for 48 h on the paracrine secretion of BM-MSCs. ELISA assay demonstrated the levels of VEGF (A), bFGF (B), IGF-1 (C), and HGF (D) secreted by BM-MSCs treated by HPC for 48 h with and without autophagy inhibition by 3-MA and Atg7 siRNA. Data are expressed as means ± SEM; *n* = 5; *p < 0.05. (TIF 663 kb)

Additional file 3: Figure S3. Autophagy modulated the adverse effect of HPC for 48 h on BM-MSCs. (A) Representative TUNEL images of BM-MSCs treated by HPC for 48 h with or without autophagy inhibition by 3-MA and Atg7siRNA. Scale bars = 20 μm. (B) The quantification of the apoptotic BM-MSCs in all groups. Data are expressed as means ± SEM; *n* = 5; *p < 0.05. (TIF 2401 kb)

Additional file 4: Figure S4. Effect of 3-MA and Atg7 siRNA on the autophagy of BM-MSCs with HPC. (A) Representative immunofluorescence images of GFP-LC3 (green fluorescence) and DAPI (blue fluorescence) in BM-MSCs with HPC and the autophagy inhibitor 3-MA and Atg7 siRNA, respectively. Scale bars = 20 μm. (B) Quantification of autophagy flux was presented as the percentage of BM-MSCs with punctate LC3 in all groups. *p < 0.05. (TIF 1978 kb)

Additional file 5 Figure S5. Evaluation of the survival of transplanted BM-MSCs with different HPC protocols. (A) Representative longitudinal BLI spatiotemporally tracked BM-MSCs (top row, *n* = 10), HPC 24 h MSCs (second row, *n* = 10), and HPC 48 h MSCs (third row, *n* = 10). Color scale bar values are in photons/s/cm^2^/sr. (B) Quantitative analysis of Fluc optical signals on fixed regions of interest (ROI). (TIF 3197 kb)

## Abbreviations

αMEM: Alpha-modified Eagle’s medium
3MA: 3-Methyladenine
ANOVA: Analysis of variance
ATG: Autophagy associated gene
bFGF: Basic fibroblast growth factor
BLI: Bioluminescence imaging
BM-MSC: Bone marrow mesenchymal stem cell
DAPI: 4,6-Diamidino-2-phenylindole
DMEM: Dulbecco’s modified Eagle’s medium
eGFP: Enhanced green fluorescent protein
ELISA: Enzyme-linked immunosorbent assay
FBS: Fetal bovine serum
FCS: Fetal calf serum
Fluc: Firefly luciferase
FS: Fractional shortening
H/SD: Hypoxia/serum deprivation
HGF: Hepatocyte growth factor
HPC: Hypoxic preconditioning
^HPC^MSC: Hypoxic preconditioned (bone marrow) mesenchymal stem cell
IGF: Insulin-like growth factor
LAD: Left anterior descending
LVEDD: Left ventricular end-diastolic diameter
LVEDV: Left ventricular end-diastolic volume
LVEF: Left ventricular ejection fraction
LVESD: Left ventricular end-systolic diameter
LVESV: Left ventricular end-systolic volume
MI: Myocardial infarction
MTT: 3-(4,5-Dimethylthiazol-2-yl)-2,5- diphenyltetrazolium bromide
^NPC^MSC: Normal preconditioned (bone marrow) mesenchymal stem cell
OD: Optical density
PBS: Phosphate-buffered saline
siRNA: Small interfering RNA
TUNEL: Terminal deoxynucleotidyl transferase-mediated nick-end labeling
VEGF: Vascular endothelial growth factor
