# Supplementary figures and images for: Autophagy mediates the beneficial effect of hypoxic preconditioning on bone marrow mesenchymal stem cells for the therapy of myocardial infarction
Source: Stem Cell Res Ther. 2017 Apr 18;8:89. doi: 10.1186/s13287-017-0543-0 (PMC5395756; doi:10.1186/s13287-017-0543-0)

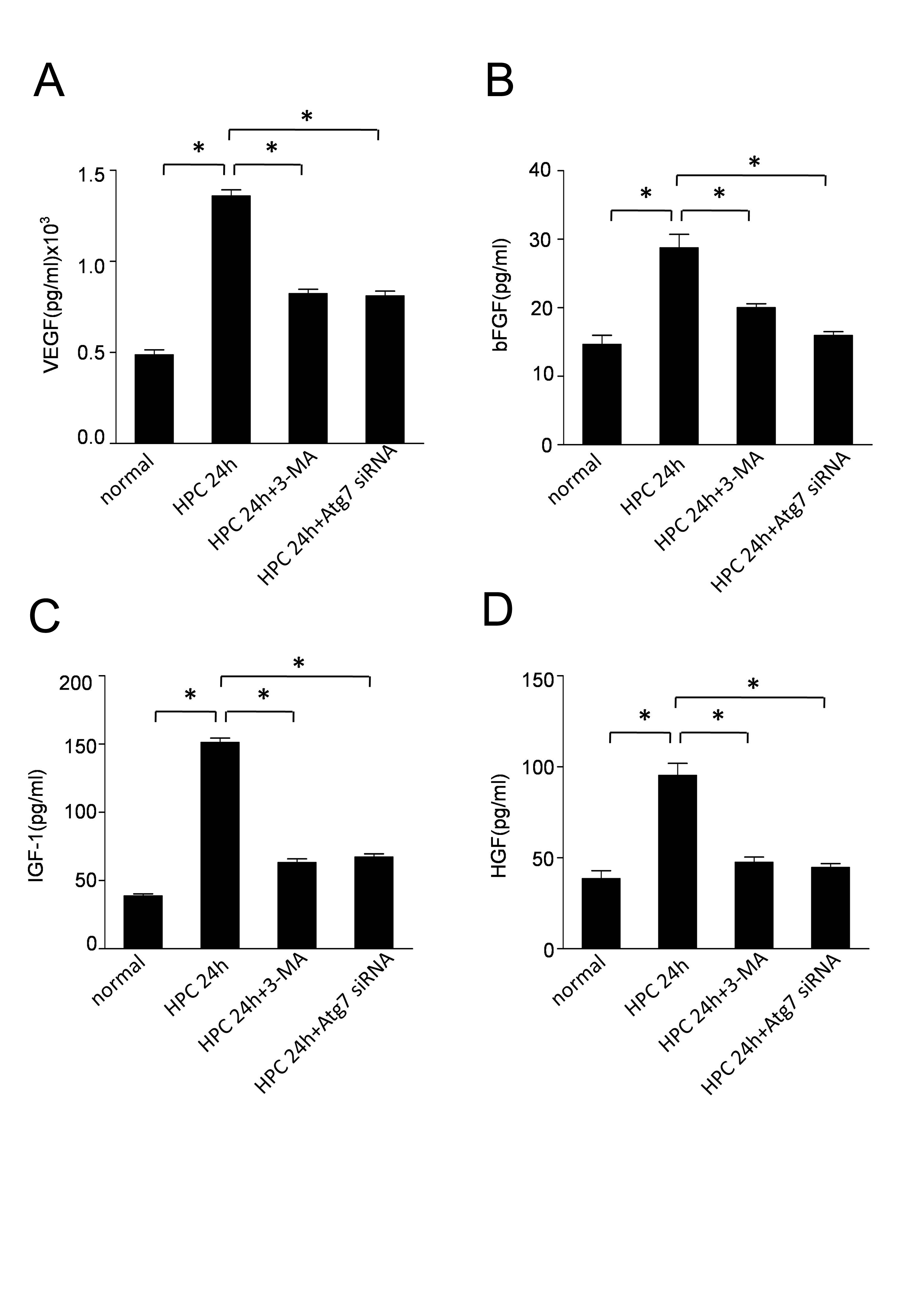

Supplement: Supplementary file 1 — The effect of autophagy inhibition on the paracrine secretions of BM-MSCs treated by HPC for 24 h. ELISA assay demonstrated the levels of VEGF (A), bFGF (B), IGF-1 (C), and HGF (D) secreted by BM-MSCs treated by HPC for 24 h with and without autophagy inhibition by 3-MA and Atg7 siRNA. Data are expressed as means ± SEM; n = 5; *p < 0.05. (TIF 626 kb) [file 13287_2017_543_MOESM1_ESM.tif]

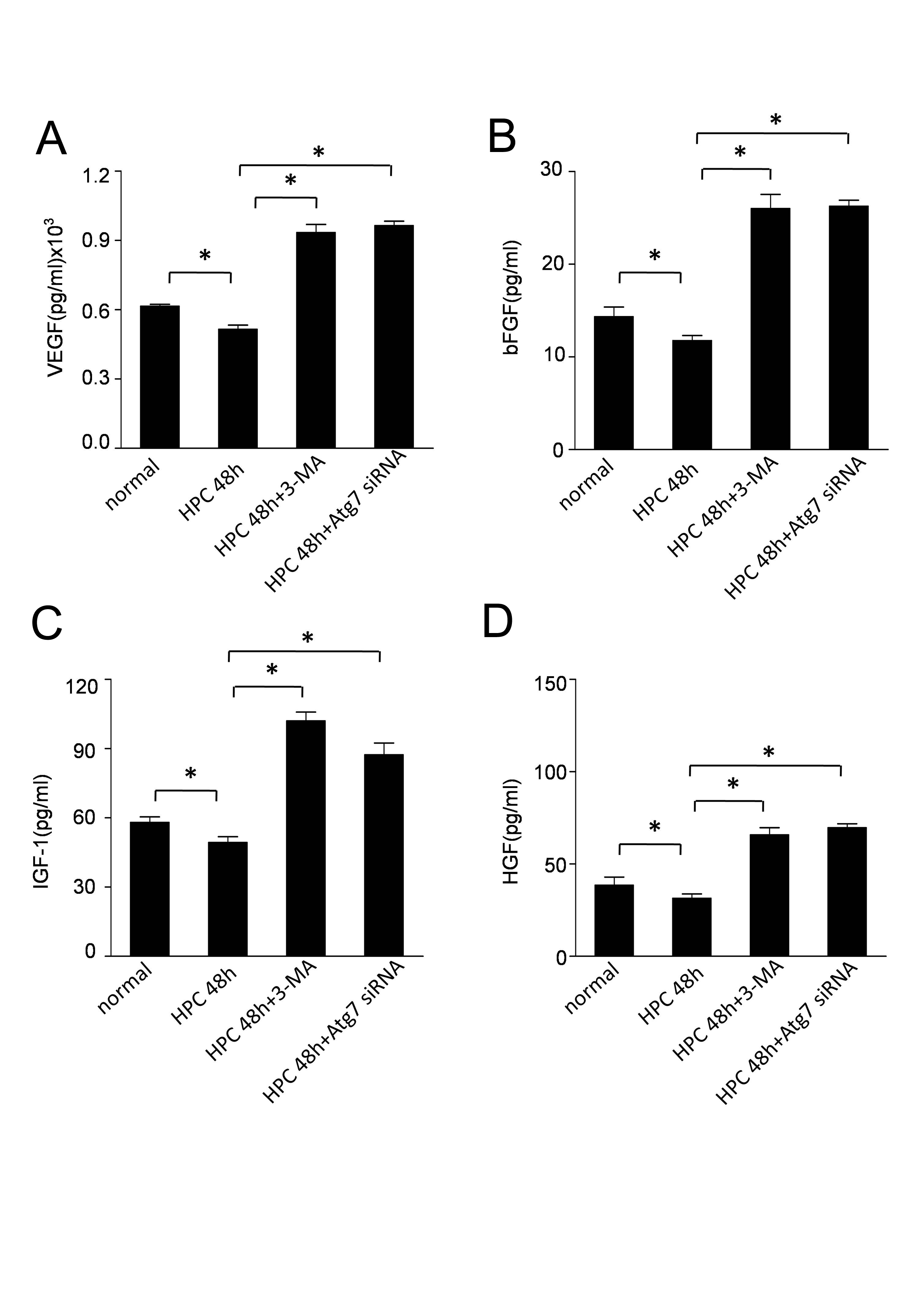

Supplement: Supplementary file 2 — Effect of HPC for 48 h on the paracrine secretion of BM-MSCs. ELISA assay demonstrated the levels of VEGF (A), bFGF (B), IGF-1 (C), and HGF (D) secreted by BM-MSCs treated by HPC for 48 h with and without autophagy inhibition by 3-MA and Atg7 siRNA. Data are expressed as means ± SEM; n = 5; *p < 0.05. (TIF 663 kb) [file 13287_2017_543_MOESM2_ESM.tif]

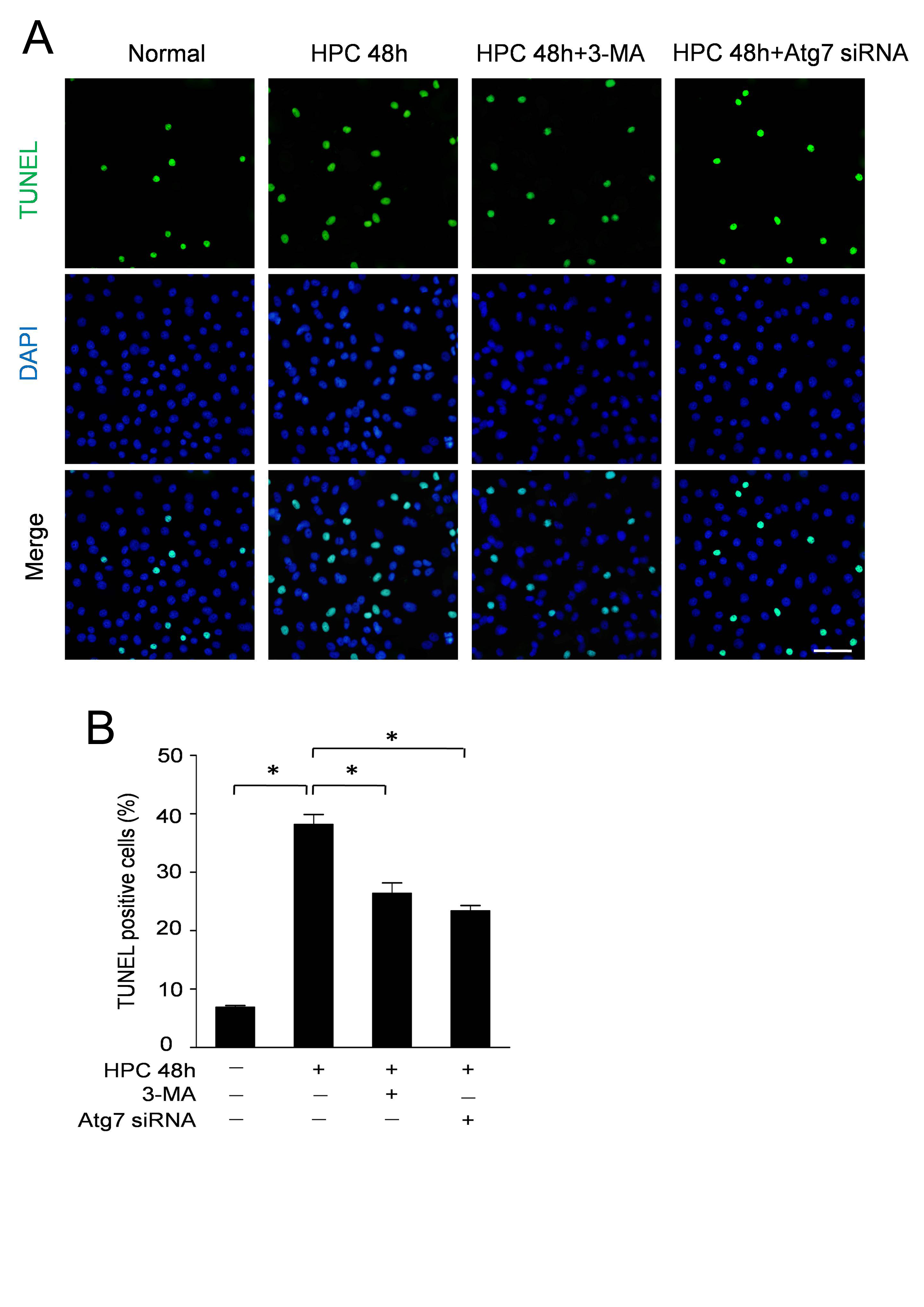

Supplement: Supplementary file 3 — Autophagy modulated the adverse effect of HPC for 48 h on BM-MSCs. (A) Representative TUNEL images of BM-MSCs treated by HPC for 48 h with or without autophagy inhibition by 3-MA and Atg7siRNA. Scale bars = 20 μm. (B) The quantification of the apoptotic BM-MSCs in all groups. Data are expressed as means ± SEM; n = 5; *p < 0.05. (TIF 2401 kb) [file 13287_2017_543_MOESM3_ESM.tif]

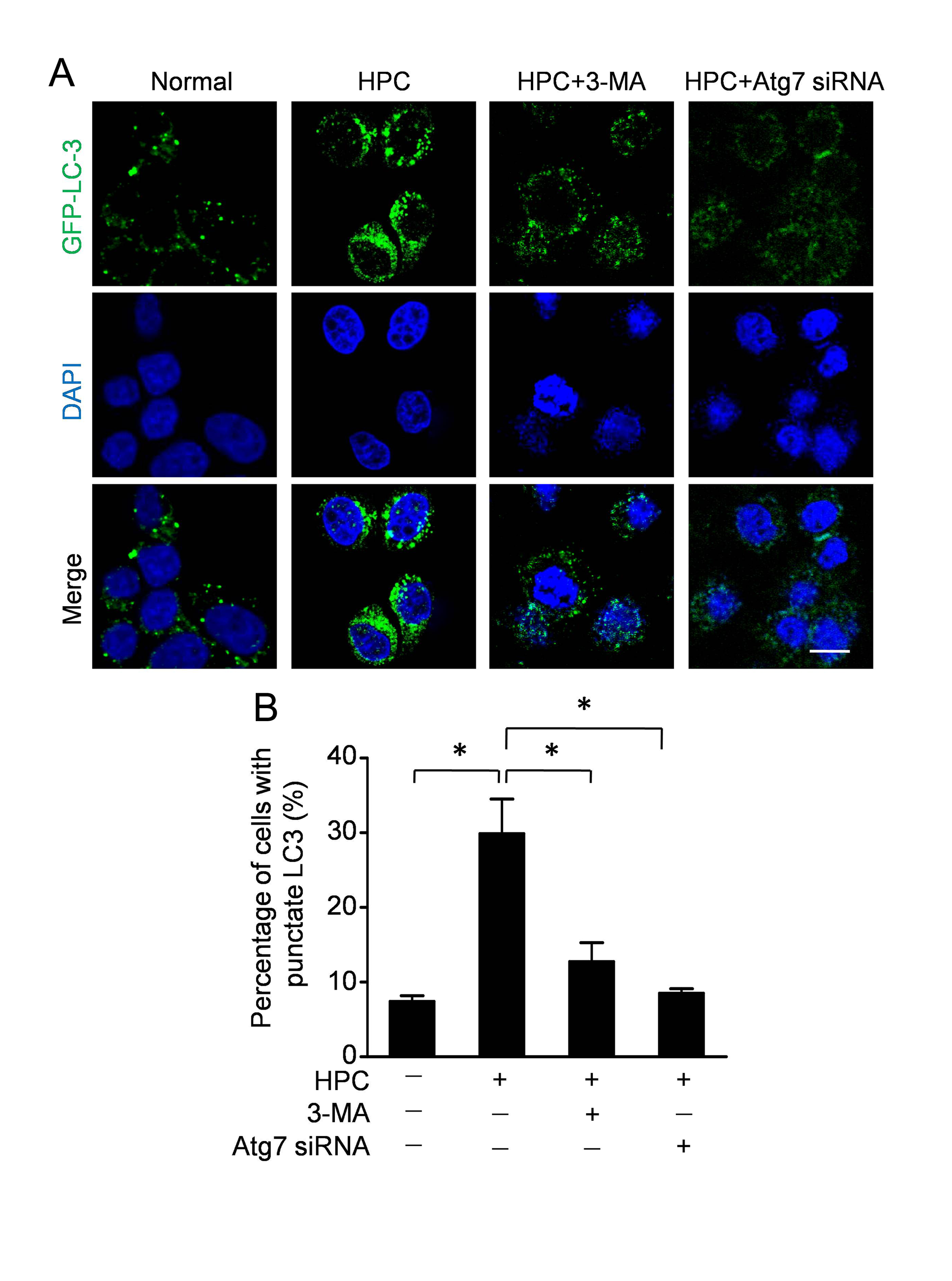

Supplement: Supplementary file 4 — Effect of 3-MA and Atg7 siRNA on the autophagy of BM-MSCs with HPC. (A) Representative immunofluorescence images of GFP-LC3 (green fluorescence) and DAPI (blue fluorescence) in BM-MSCs with HPC and the autophagy inhibitor 3-MA and Atg7 siRNA, respectively. Scale bars = 20 μm. (B) Quantification of autophagy flux was presented as the percentage of BM-MSCs with punctate LC3 in all groups. *p < 0.05. (TIF 1978 kb) [file 13287_2017_543_MOESM4_ESM.tif]

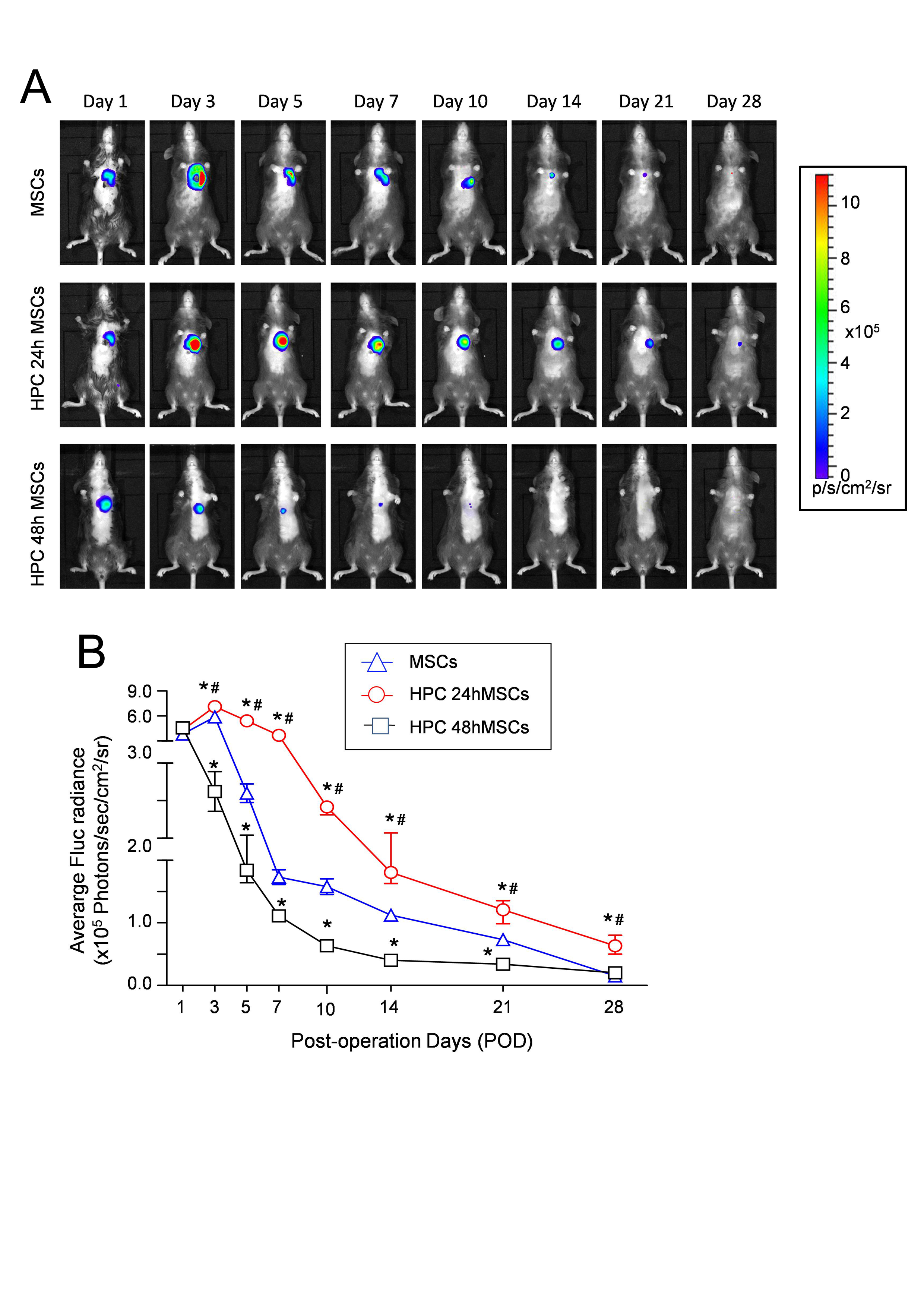

Supplement: Supplementary file 5 — Evaluation of the survival of transplanted BM-MSCs with different HPC protocols. (A) Representative longitudinal BLI spatiotemporally tracked BM-MSCs (top row, n = 10), HPC 24 h MSCs (second row, n = 10), and HPC 48 h MSCs (third row, n = 10). Color scale bar values are in photons/s/cm2/sr. (B) Quantitative analysis of Fluc optical signals on fixed regions of interest (ROI). (TIF 3197 kb) [file 13287_2017_543_MOESM5_ESM.tif]
